# Benign metastasizing leiomyoma: A review of current literature in respect to the time and type of previous gynecological surgery

**DOI:** 10.1371/journal.pone.0175875

**Published:** 2017-04-20

**Authors:** Edyta Barnaś, Mariusz Książek, Renata Raś, Andrzej Skręt, Joanna Skręt- Magierło, Ewa Dmoch- Gajzlerska

**Affiliations:** 1 Institute of Obstetrics and Emergency Medicine, Medical Faculty, University of Rzeszow, Rzeszow, Poland; 2 Clinical Department of Pathology, Frederick Chopin Clinical Provincial Hospital No 1, Rzeszow, Poland; 3 Obstetrics and Gynecology Clinic, Frederick Chopin Clinical Provincial Hospital No 1 Rzeszow, University of Rzeszow, Rzeszow, Poland; 4 Faculty of Health Sciences, Gynaecological and Obstetrics Department, Medical University of Warsaw, Warsaw, Poland; Duke University, UNITED STATES

## Abstract

**Introduction:**

Benign metastasizing leiomyoma (BML) is a rare disorder that affects women with a history of uterine leiomyoma, which is found to metastasise within extrauterine sites. The aetiology of BML remains unexplained. Because BML is rare, and most publications contain descriptions of single cases, no statistically determined time relations were found between the primary and secondary surgeries, which may have aetiological implications.

**Objectives:**

To determine age before BML surgery, age during diagnosis of BML, type of prior surgery, and location of metastasis based on the literature.

**Methods:**

A systematic review of four databases (Medline/PubMed, Embase, Web of Science, and Cochrane) covering articles published from 1 January 1965 to 10 April 2016. The inclusion criteria were full-text articles in English and articles containing case reports. Articles in languages other than English (39), articles containing incomplete data (14), i.e. no information regarding the time of surgery and/or the site of metastasis, articles bereft of case studies (25), and articles with access only to summaries, without access to the complete text (10) were excluded. Of 321 titles identified, only 126 articles met the aforementioned criteria.

**Results and conclusions:**

The mean age during primary surgery and BML diagnosis was 38.5 years and 47.3 years, respectively. The most common surgery was total hysterectomy. The most frequent site of metastasis was the lungs; other organs were affected less frequently.The site of metastases and their number were not related to the longer time span between the patient’s initial surgery and occurrence of metastasis. The analysed data, such as the age during primary surgery, age during BML diagnosis, site and type of metastasis, do not provide us a clear answer. Thus, BML pathogenesis is most probably complex in nature and requires further multidirectional research.

## Introduction

Benign metastasizing leiomyoma (BML) is a rare disorder that affects women with a history of uterine leiomyoma, which is found to metastasise within extrauterine sites.The disease develops as a proliferation of multiple nodules composed of smooth muscle cells.The most frequent site of metastasis is the lungs, although other areas may also be affected as well, including some atypical locations, e.g. the heart or spinal cord.Steiner (1939) was first to describe this disease in detail.He published a report of a patient who died from the effects of extensive pulmonary metastases of benign-appearing leiomyomas, which were histologically identical to the multiple leiomyomas in the uterus [1].

The majority of case study authors demonstrate the time relation between the patient’s primary surgery and BML onset.To the best of our knowledge, the literature on this subject describes only 10 cases of BML in women who have not undergone prior surgery [2–11].

Because BML is rare, and most publications contain descriptions of single cases, no statistically determined time relations were found between the primary and secondary surgeries.Few case report or review authors have stated that the estimated time from the initial surgery to time of BML diagnosis, which was speculated to be approximately 10 to 15 years [2,12,13]. No associated literature or any review publications to date have analysed the compiled date, with respect to the site of metastasis and type of primary surgery. Therefore, we decided that the characteristics of those relations were to be the focus of our investigation to determine their importance in the aetiopathogenesis of the disease. The entire literature concerning the subject of BML available in various medical databases was analysed to achieve this.The aim of the thesis was defined as the following:

1. To determine the following based on the literature data:
  - Age of female patients with BML when the primary surgery was performed.
  - Age of female patients during BML diagnosis.
  - Time between the primary and secondary surgeries;
2. To rank these parameters for the type of the primary surgery and the site of metastasis; and
3. To determine the importance of such data, with respect to the type of aethiopathogenesis of BML.

## Materials and methods

### Search strategy

The analysis includes academic publications that contain the term “benign metastasizing leiomyoma”, and the search was performed in four databases:Medline/PubMed, Embase, Web of Science, and Cochrane. Our research date was 10 April 2016. The literature was compiled from April to May 2016.

As an initial step, we found 321 hits for a broad search string [metastasizing* OR leiomyoma*], and 214 hits were found when the searched term was reduced to “benign metastasizing leiomyoma”.The researched articles were published between 1965 and 2016.

The inclusion criteria were the following:

➢ Full-text publications in English
➢ Articles containing case reports

The exclusion criteria were the following:

➢ Articles in languages other than English
➢ Articles containing incomplete data, i.e. no information regarding the time of surgery and/or the site of metastasis
➢ Articles bereft of case studies
➢ Article with access only to summaries, without access to the complete text.

**Fig 1:**
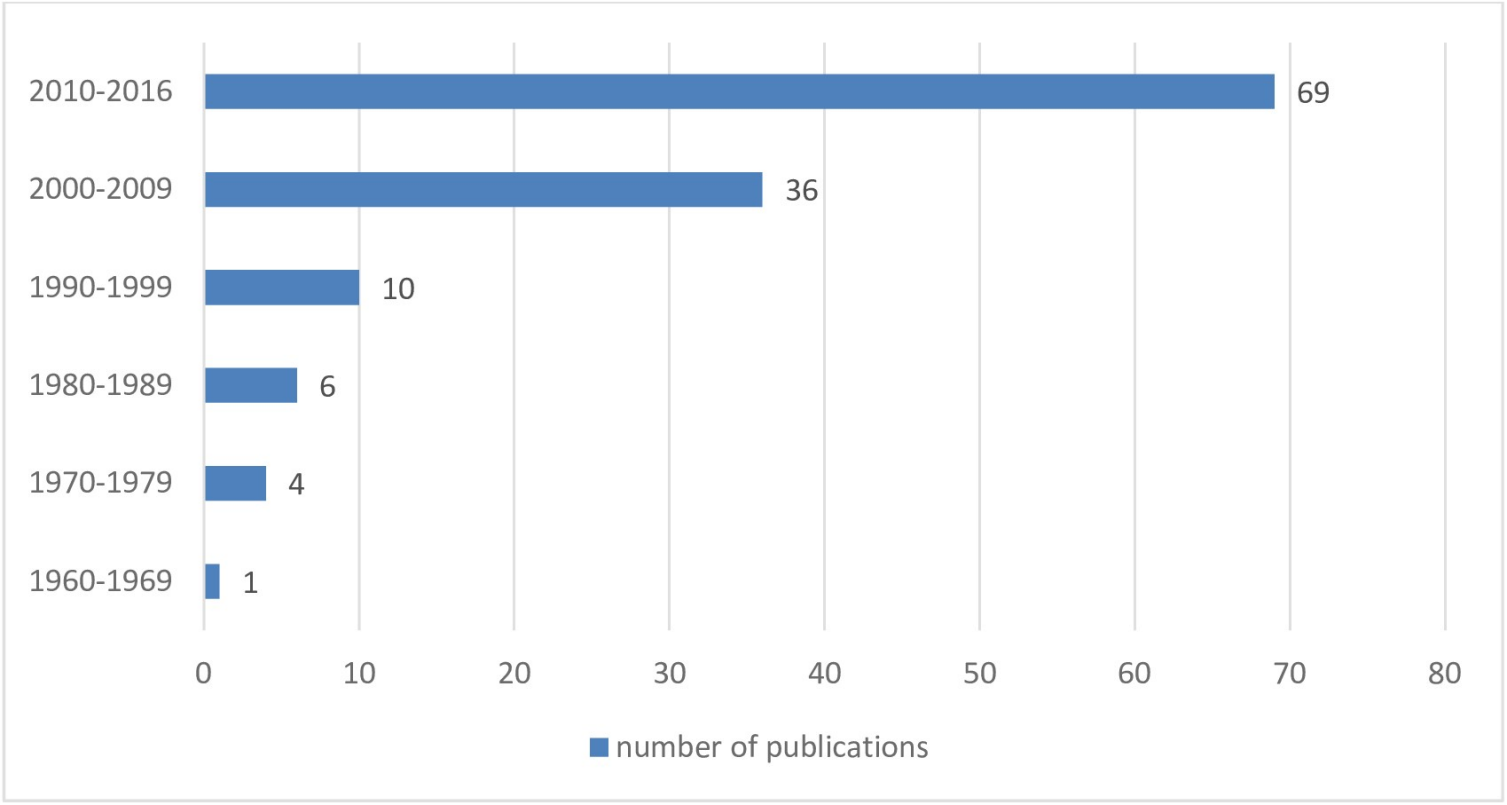
Number of analysed publications on BML which meet the inclusion criteria, time frame from 1960 to 10 April 2016.

The aforementioned criteria were met by 126 articles, and the time frame from 1960 to 2016 is presented in Fig 1.

Articles included in the analysis based on the inclusion criteria was shown in S1 File.

### Data analyses

Statistica 10.0 software was used to analyse the data, and the tests performed included chi-square, analysis of variance, Kruskal–Wallis analysis, and Mann–Whitney U test.A statistical significance level at p < 0.05 was adopted.

## Results

**Fig 2:**
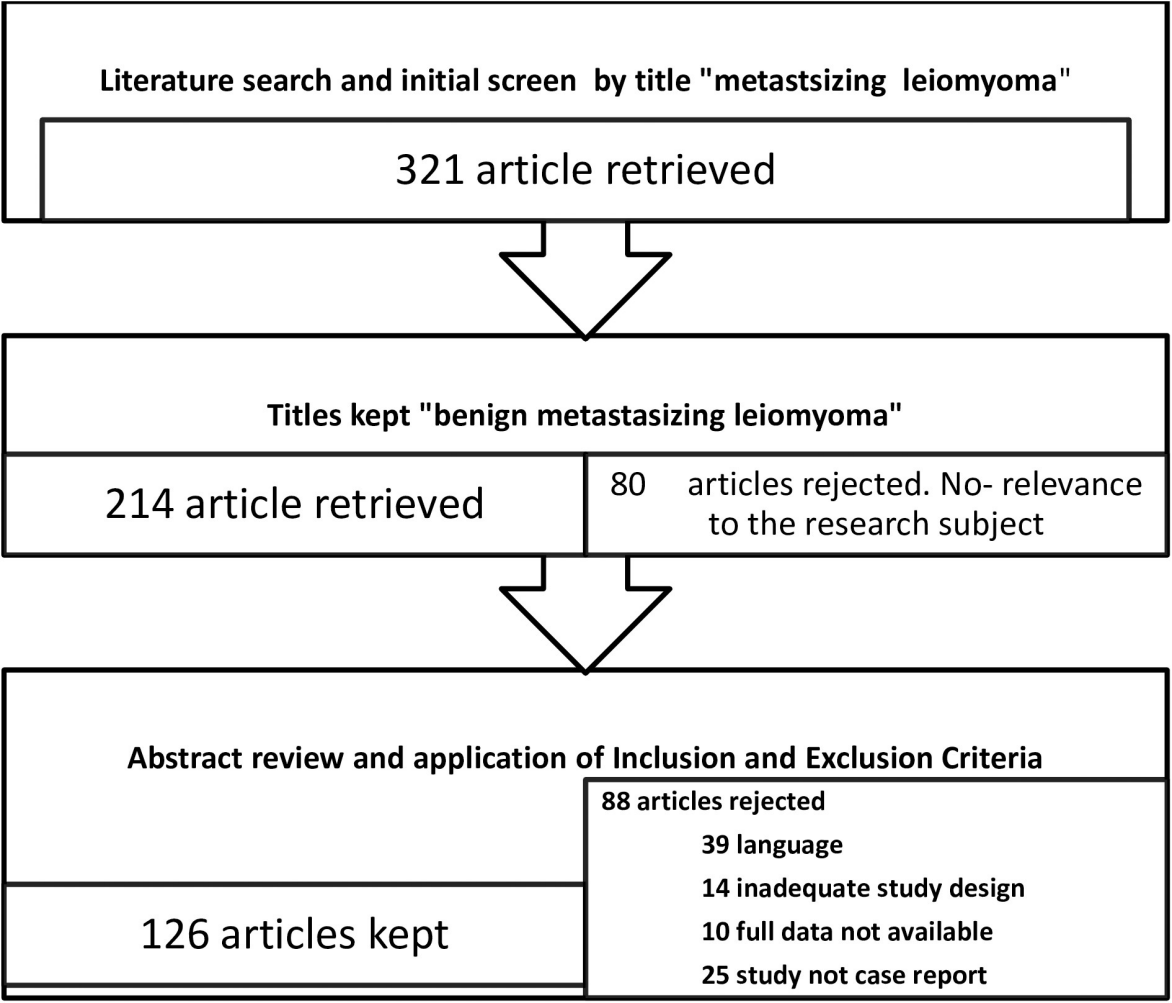
Summary of retrieval and review of articles on benign metastasizing leiomyoma, 1965–2016.

From a group of 214 selected articles, 126 were included in the final analysis, of which 161 case studies were found to provide comprehensive data, such as the patient age during BML diagnosis, age during the primary surgery, and site of metastasis (Fig 2).

Analysis of the type of the primary surgery demonstrated that conservative myomectomy was performed in the youngest patients. In addition, the mean age of the patients with BML who underwent surgery showed that this procedure was performed in the youngest patients (Table 1A). The analysis of the site of metastasis and mean age during the primary and BML surgery did not exhibit any significant differences between the groups (Table 1B).

**Table 1:** Analysis of the relationship between selected variables.

Variables		Mean age during primary surgery (mean±SD)	Statistical significance		Mean age during BML surgery (mean±SD)	Statistical significance	
**A**	1) Myomectomy	33.29± 6.31	1 vs 2	0,00576	42.84±8.01	1 vs 2	0,00508
**type of surge-ry**	2) Subtotal hysterectomy	44.00±7.23	2 vs 3	0,09595	51.67±2.73	2 vs 3	0,15880
	3) Hysterectomy	39.03±9.23	3 vs 1	0,00087	48.33±10.48	3 vs 1	0,00158
**B**	4) Lungs	37.71±9.08	4 vs 5	0,114006	47.37±10.24	4 vs 5	0,504546
**site of meta-stasis**	5) Other^b^	42.75±14.94	5 vs 6	0,438110	46.41±10.41	5 vs 6	0,466855
	6) Lungs + other	39.22±9.57	6 vs 4	0,700107	48.44±8.14	6 vs 4	0,557527

^b)^Other- spinal, breast, pleurae, brain, rib and vertebral, appendix, parametria, heart, vessels, skeletal, muscle, soft tissue, lymph node, and retroperitoneal

The mean age during the primary surgery was 38.5 years in the entire group of 160 cases, whereas the mean age for BML diagnosis was 47.3 years (Table 2).

**Table 2:** Group descriptive statistics.

Group data	n	Mean	Min	Max.	Variation	Standard deviation	Coefficient of variation
**Age during myoma surgery (years)**	161	38.5	18	72	80	8.99	23.57
**Age during BML diagnosis (years)**	161	47.3	22	77	101.65	10.02	21.32

The patients’ age during the primary surgery correlated with the patients age during BML diagnosis, except subtotal hysterectomy (STH) cases (Table 3A). In addition, the mean age of the female patients during the initial surgery correlated with the mean age during diagnosis in the majority of patients with BML, with metastasis particularly to the lungs (Table 3B).

**Table 3:** Results of the analysis of regression, in general, and in groups determined by the type of surgery and the site of metastasis.

**A**	**Variables**	**Measure**	**Total**	**Myomectomy**	**Hysterectomy**	**Subtotal myomectomy**
	**Age during surgery to age during BML diagnosis (years)**	R	0.69	0.64	0.68	−0.04
		p	0.0000	0.0000	0.0000	0.939
		R^2^	0.47	0.41	0.46	0.0016
		Y	Y = 0.77x + 17.9	Y = 0.81x+15.81	Y = 0.77x+18.01	Y = -0.2x+52.33
**B**	**Variables**	**Measure**	**Total**	**Lungs**	**Other**	**Lungs and other**
	**Age during surgery to age during BML diagnosis (years)**	R	0.71	0.52	0.72	0.45
		p	0.001	0.0000	0.0000	0.225
		R2	0.49	0.27	0.85	0.20
		Y	Y = 0.77x + 18.19	Y = 0.57x + 9.97	Y = 0.89x + 10.87	Y = 0.38x + 33

R- Pearson’s correlation coefficient. R2 –coefficient of determination.—statistical significance level. Y- regression model.

No significant differences were found in the relationship between the time of the primary surgery and BML diagnosis, with respect to the type of surgery (Table 4A).Furthermore, no significant differences were found in the relationship between the time of the primary surgery and BML diagnosis, with respect to the site of metastasis (Table 4B).

**Table 4:** Descriptive statistics of the time from the primary surgery to BML diagnosis, grouped based on the type of surgery and the site of metastasis.

**A**	**Type of surgery**	**n**	$\left(\bar{X}\pm \sigma )\;(\bar{x}\pm \sigma \right)$	**σ**^**2**^***σ***^**2**^	**Min.**	**Max.**	**Q25**	**Me**	**Q75**	p = 0.7481
	Myomectomy	32	(9.54±6.27)	39.3	0	30	5	10	12	
	Hysterectomy	122	(9.59±7.57)	57.3	0	31	4	9	14	
	Subtotal hysterectomy	7	(7.67±7.84)	61.5	0	21	0	7	11	
	Total	161	(9.51±7.31)	53.43	0	31	4	9	14	
**B**	**Site of metastasis**	**n**	$\left(\bar{X}\pm \sigma )\;(\bar{x}\pm \sigma \right)$	**σ**^**2**^***σ***^**2**^	**Min.**	**Max.**	**Q25**	**Me**	**Q75**	p = 0.0570
	Lungs	128	(9.95±7.14)	50.99	0	31	5	10	14	
	Other	24	(6.56±7.28)	52.98	0	31	2	4	11	
	Lungs and other	9	(9.22±9.38)	87.94	0	21	0	4	20	
	Total	161	(9.42±7.34)	53.91	0	31	4	9	14	

**Fig 3:**
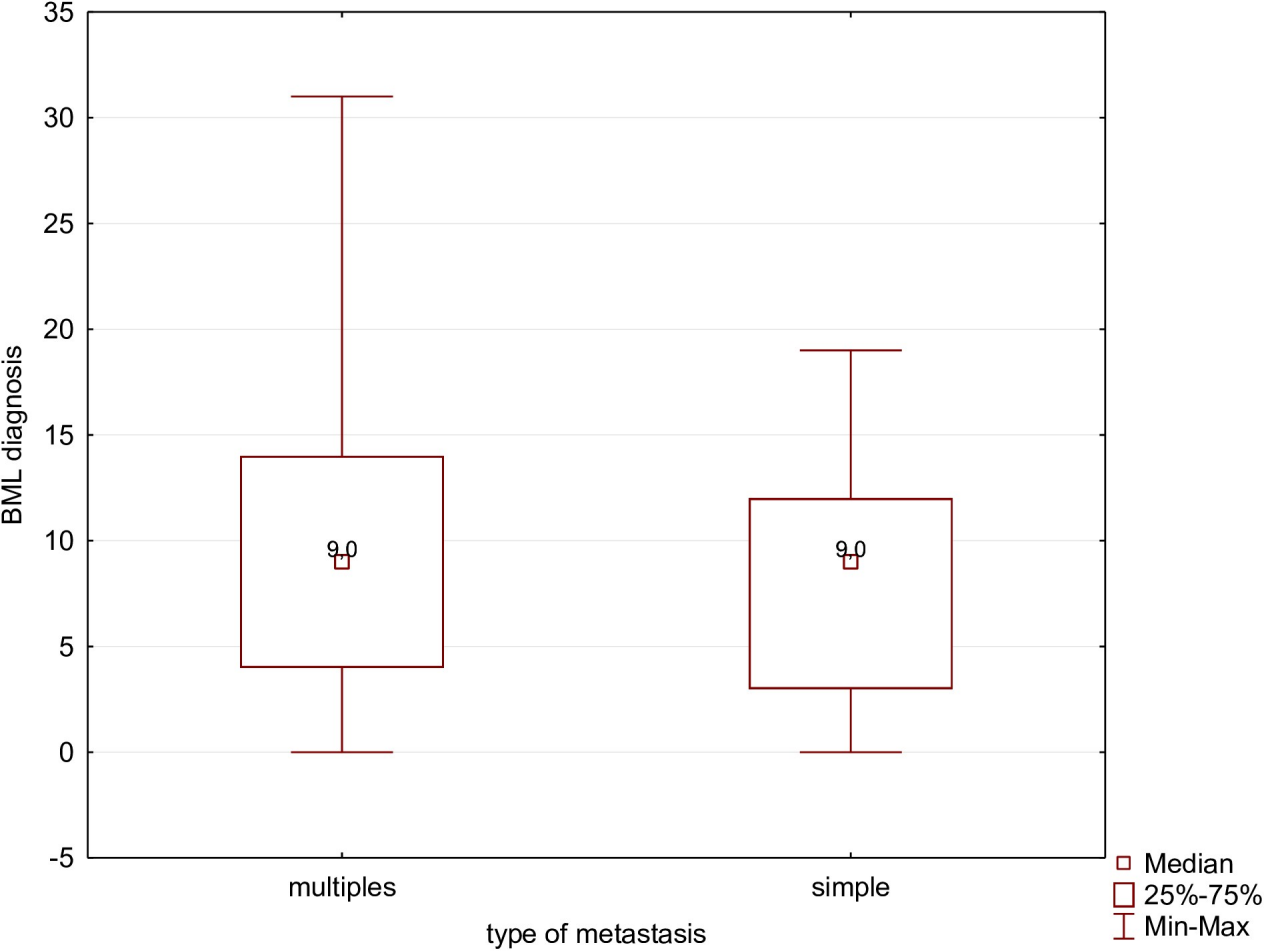
BML diagnosis and the type of metastasis.

The analysis of the relationship between BML diagnosis and characteristics (single site versus multiple sites) of the diagnosed metastases did not show any significant differences (p = 0.737517) (Fig 3).

## Discussion

This work is a pioneering endeavor.Although case reports are available in literature reviews, they have substantially narrow time scopes [2,3,12,13,14–29].Moreover, no articles demonstrate the time relationships between parameters, i.e. the primary surgery and BML diagnosis. Therefore, the results of this study cannot be compared with the works of other authors.

The overall incidence of BML after leiomyoma is unknown, as well as the incidence of BML after various types of surgery. Therefore, the risk associated with the type of operation cannot be determined.

The occurrence of metastatic leiomyomas, in all types of surgeries, does not substantiate the claim that any particular type of surgery predisposes their occurrence,particularly because cases have occured wherein BML was diagnosed in women who had not undergone a previous uterine myoma surgery [2–11]. However, another suggested theory for BML is peritoneal seeding after myomectomy or hysterectomy for uterine leiomyoma.Fragments of uterine leiomyoma may possibly implant and proliferate when accidentally left inside the peritoneum after laparotomy or after laparoscopic morcellation. Laparoscopic morcellation is a relatively new technique employed for approximately 10 years, in the analyzed studies there are no exact data on the description of its long term follow up including BML.

The current literature on the subject does not mention patients who were previously treated with laparoscopy, and there are only limited records of benign leiomyoma implants to the peritoneum occuring after such procedures [30].This independence of the disease, from the type of primary surgery, argues against the haematogenous spread of a uterine leiomyoma. In addition, the time duration between the primary surgery and BML occurence argues against the haematogenous theory of the disease, but may support the metaplasia theory.

Metaplastic transformation of the coelomic epithelium may explain BML in almost any place where mesothelial mesenchyme exists.These tumours probably originate from subcoelomic mesenchymal cells, which differentiate from the process of metaplasia into the myofibroblasts [21].

The study included completely benign leiomyoma, diagnosed after the primary surgery. A few studies have documented that the indication for the primary surgery was: abnormal bleeding associated with abdominal pain or a change detected in USG during a routine gynecological examination [6, 18, 23, 26, 29].

The mean age of the patient during the initial operation was 38.5 years old. The diagnosis was at 47.3 years, at this age most symptomatic metastases were detected, therefore, it cannot be ruled out that microscopic metastases had been present earlier. Miller et al. reported the mean age at diagnosis BML was 54.1 years [18].

Detection of metastases in 47.5% of cases was the result of reported complaints, such as cough, dyspnoea, shortness of breath, chest pain and pneumothorax. Conversely, the detection of any change, during a follow-up was less frequent, with 35.6% of cases. On the other hand, a random detection during the preparation for surgery was observed in 8.75% of. The disease is so rare that it is unreasonable to perform a screening test in all women undergoing surgery because of leiomyoma.

Metastatic sites were found in various locations, among others: spinal, breast, pleurae, brain, rib and vertebral, appendix, parametria, heart, vessels, skeletal, muscle, soft tissue, lymph node, and retroperitoneal [3,25,31–40] The majority of them were bilateral, with an average size from 2mm×3 mm to 2.7 cm×4.4 cm. 53.75% of the patients exhibited multiple metastasis [2,5,7,8,12,13,15,16,17,20,41].

BML has been recently suggested as the result of monoclonal, haematogenous spread of benign-appearing uterine leiomyoma.The morphology, molecular and immunohistochemical features are characteristics for benign neoplasms despite the metastatic potential.

In review study, microscopic examination of haematoxylin and eosin slides has demonstrated the characteristic features of smooth muscle cell differentiation, which was also confirmed by immunohistochemistry smooth muscle actin positivity.Additional immunohistochemistry Ki67 showed a low tumour cell proliferation index, which favors a benign behavior [3,4,7,8,11–14,17,18,20–22,27,31–37, 41–55].

The microscopic criteria for the diagnosis of a benign leiomyoma, smooth muscle tumours with atypical features, as well as malignant leiomyosarcoma are well defined. It was assumed that there were no features of malignancy (necrosis, increased mitotic activity, marked cellular pleomorphism) within the primary surgical specimens since they were not mentioned in the original diagnosis.

Also, by definition, the BML specimens showed no microscopic features of malignancy, and as previously mentioned, in the majority of described cases, they closely resembled the histological benign features of primary uteral leiomyomas.

Although there are few, reports dealing with the genetic aspect of the subject, most of them confirm—the monoclonality of the primary benign smooth muscle tumour and BMLin molecular studies.

Hypothesisof genomic imbalance, such as the rearrangement of HMGA1 (6p21), shows the association of such changes with BML [20].

Conventional cytogenetic studies have provided valuable insight, regarding the histopathogenesis of numerous mesenchymal neoplasms.Only scant pulmonary BML has been previously characterised karyotypically.The studies confirmed the presence of karyotypic aberrations in 56% of cases of benign leiomyomas [56–58].Lee et al. analysed reports describing balanced translocations, including t(12;14) (q14-15;q23-24), t(12;14)(q13-15;q32), and t(1;2)(p36;p24), which have been most commonly observed among uterine leiomyomas [31].However, Nucci et al. described consistent chromosomal aberrations (19q and 22q terminal deletions in all 5 cases) in BML cases and suggested that BML is a genetically distinct entity [59].Lee et al. concluded that BML may comprise a heterogenous group of tumours in terms of their malignant potential and pathogenetic mechanisms.However, in their described case, significant genetic abnormalities were shared by both lesions from the uterus and lungs.No further growth was observed in the number and size of the remaining pulmonary nodules after hysterectomy, and this supports a transportation theory of BML [31].Similar conclusions have been shown by Bowen et al. [60]. Their study, supported by conventional karyotypic, fluorescence in situ hybridization, and whole genome SNP array analysis, suggests that both the deep soft tissue leiomyoma and pleuropulmonary BML were derived from the same abnormal clone and are genetically related to uterine leiomyoma.Patton et al. assessed the variable length of the polymorphic CAG repeat sequence within the human androgen receptor gene on pulmonary and uterine lesions from two informative patients and found identical patterns of androgen receptor allelic inactivation, which indicated that the lesions were clonal.The telomere length measured by fluorescence in situ hybridization in pulmonary leiomyomas, of all three patients, were either long or very long, and were identical to the uterine counterparts, indicating that significant telomere shortening is not a crucial step for developing metastases.Their evidence supports the notion that BML is clonally derived from benign-appearing uterine leiomyomas [61].He demonstrated that telomere shortening was not responsible for metastatic spread.

## Conclusions

The mean time from the primary surgery to BML diagnosis was 8.8 years.The age at which BML occurs was predominantly within the perimenopausal period.The most common surgery was total hysterectomy. The most frequent site of metastasis was the lungs; other organs were less frequently affected.The site of metastases and their number were not related to the longer time span between the patient’s initial surgery and occurrence of metastasis. The analysed data, such as the age during the primary surgery, age during BML diagnosis, site and type of metastasis, do not provide us a clear answer. Thus, BML pathogenesis is most probably complex in nature and requires further multidirectional research.

## Supporting information

S1 Checklist PRISMA 2009 checklist. (DOC)

S1 File Articles included in the analysis based on the inclusion criteria. (DOCX)
