# Supplementary material for: Benign metastasizing leiomyoma: A review of current literature in respect to the time and type of previous gynecological surgery
Source: PLoS One. 2017 Apr 20;12(4):e0175875. doi: 10.1371/journal.pone.0175875 (PMC5398563; doi:10.1371/journal.pone.0175875)
Supplement: S1 File — (DOCX) [file pone.0175875.s002.docx]

**S1 File. Articles included in the analysis based on the inclusion criteria**

1. Abell MR, Littler ER. Benign metastasizing uterine leiomyoma. Multiple lymph nodal metastases. Cancer. 1975 Dec;36(6):2206-13.
2. Aboualfa K, Calandriello L, Dusmet M, Ladas G, Hansell DM, Nicholson AG. Benign metastasizing leiomyoma presenting as cystic lung disease: a diagnostic pitfall. Histopathology. 2011 Oct;59(4):796-9. doi: 10.1111/j.1365-2559.2011.03952.x.
3. Abramson S, Gilkeson RC, Goldstein JD, Woodard PK, Eisenberg R, Abramson N. Benign metastasizing leiomyoma: clinical, imaging, and pathologic correlation. AJR Am J Roentgenol. 2001 Jun;176(6):1409-13.
4. Afrăsânie VA, Iacob M, Ferariu D, Demşa I, Miron L, Clement DE. Benign metastasizing leiomyoma of the lung–case report and brief review of the literature. Arch Clin Cases 2015;*2*(4):242-251.
5. Ağaçkiran Y, Findik G, Ustün LN, Aydoğdu K, Kaya S. Pulmonary Benign Metastasizing Leiomyoma: An Extremely Rare Case. Turk Patoloji Derg. 2014 Apr 9. doi: 10.5146/tjpath.2013.01217.
6. Ahmad SZ, Anupama R, Vijaykumar DK. Benign metastasizing leiomyoma - case report and review of literature. Eur J Obstet Gyn R B 2011;159(1):240-1.
7. Alessi G, Lemmerling M, Vereecken L, De Waele L. Benign metastasizing leiomyoma to skull base and spine: a report of two cases. Clin Neurol Neurosurg. 2003 Jul;105(3):170-4.
8. Anila KR, Rema P, Prahladan A, Somanathan T, Mathews A. et al. Benign Metastasizing Leiomyomas Involving Multiple Pelvic and Para- Aortic Lymph Nodes: A Case Report. J Genit Syst Disor 2013;2:3. doi:10.4172/2325-9728.1000113.
9. Arif S, Ganesan R, Spooner D. Intravascular leiomyomatosis and benign metastasizing leiomyoma: an unusual case. Int J Gynecol Cancer. 2006 May-Jun;16(3):1448-50.
10. Avedissian LS, Hnatiuk OW, Frenia DS. Benign Metastasizing Leiomyoma-An Unusual Cause of Diffuse Pulmonary Nodules. Chest. 2004;126(4):989S-990S.
11. Awonuga AO, Rotas M, Imudia AN, Choi C, Khulpateea N. Recurrent benign metastasizing leiomyoma after hysterectomy and bilateral salpingo-oophorectomy. Arch Gynecol Obstet. 2008 Oct;278(4):373-6. doi: 10.1007/s00404-008-0581-z.
12. Awonuga AO, Shavell VI, Imudia AN, Rotas M, Diamond MP, Puscheck EE. Pathogenesis of benign metastasizing leiomyoma: a review. Obstet Gynecol Surv. 2010 Mar;65(3):189-95.
13. Baboci A, Prifti E, Xhabija N, Alimehmeti M. Surgical removal of an intravenous leiomyoma with intracardiac extension and pulmonary benign metastases. Heart Lung Circ. 2014 Feb;23(2):174-6. doi: 10.1016/j.hlc.2013.10.058. Epub 2013 Oct 16.
14. Bachman D, Wolff M. Pulmonary metastases from benign-appearing smooth muscle tumors of the uterus. AJR Am J Roentgenol. 1976 Sep;127(3):441-6.
15. [Banner AS](http://www.ncbi.nlm.nih.gov/pubmed/?term=Banner%20AS%5BAuthor%5D&cauthor=true&cauthor_uid=7242600), [Carrington CB](http://www.ncbi.nlm.nih.gov/pubmed/?term=Carrington%20CB%5BAuthor%5D&cauthor=true&cauthor_uid=7242600), [Emory WB](http://www.ncbi.nlm.nih.gov/pubmed/?term=Emory%20WB%5BAuthor%5D&cauthor=true&cauthor_uid=7242600), [Kittle F](http://www.ncbi.nlm.nih.gov/pubmed/?term=Kittle%20F%5BAuthor%5D&cauthor=true&cauthor_uid=7242600), [Leonard G](http://www.ncbi.nlm.nih.gov/pubmed/?term=Leonard%20G%5BAuthor%5D&cauthor=true&cauthor_uid=7242600), [Ringus J](http://www.ncbi.nlm.nih.gov/pubmed/?term=Ringus%20J%5BAuthor%5D&cauthor=true&cauthor_uid=7242600). et al. Efficacy of oophorectomy in lymphangioleiomyomatosis and benign metastasizing leiomyoma. [N Engl J Med.](http://www.ncbi.nlm.nih.gov/pubmed/7242600) 1981 Jul 23;305(4):204-9.
16. Barbetakis N, Xenikakis T, Efstathiou A, Vlahveis M, Fessatidis I. Pulmonary benign metastasizing leiomyoma. J Cardiovasc Surg (Torino) 2003 Aug;45(4):399-400.
17. Barnes HM, Richardson PJ. Benign metastasizing fibroleiomyoma. A case report. J Obstet Gynaecol Br Commonw 1973 Jun;80(6):569-73.
18. Beck MM, Biswas B, D'Souza A, Kumar R. Benign metastasising leiomyoma after hysterectomy and bilateral salpingo-oophorectomy. Hong Kong Med J 2012;18(2):153-5.
19. Benetti-Pinto CL, Soares PM, Petta CA, De Angelo-Andrade LA. Pulmonary benign metastasizing leiomyoma: a report of 2 cases with different outcomes. J Reprod Med. 2006 Sep;51(9):715-8.
20. Bodner-Adler B, Bartl M, Wagner G. Intravenous leiomyomatosis of the uterus with pulmonary metastases or a case with benign metastasizing leiomyoma? Anticancer Research 2009;29(2):495-6.
21. Cadieux-Simard ML, Hennessey H, Small D, Daskalopoulou SS, Camlioglu E. Iatrogenic exacerbation of pulmonary arteriovenous malformation in a patient with benign metastasizing leiomyoma. Respirol Case Rep 2014 Mar;2(1):15-7. doi: 10.1002/rcr2.34. Epub 2013 Dec 11.
22. Cai A, Li L, Tan H, Mo Y, Zhou Y. Benign metastasizing leiomyoma. Case report and review of the literature. Herz 2014 Nov;39(7):867-70. doi: 10.1007/s00059-013-3904-1. Epub 2013 Aug 2.
23. Canzonieri V, D'Amore ES, Bartoloni G, Piazza M, Blandamura S, Carbone A. Leiomyomatosis with vascular invasion. A unified pathogenesis regarding leiomyoma with vascular microinvasion, benign metastasizing leiomyoma and intravenous leiomyomatosis. Virchows Archiv 1994;425(5):541-5.
24. Chan JW, Law WL, Cheung SO, Lee MP, Ng CK, Lee S. et al. Benign metastasising leiomyoma: a rare but possible cause of bilateral pulmonary nodules in Chinese patients. Hong Kong Med J 2005;11(4):303-6.
25. Chen S, Liu RM, Li T. Pulmonary benign metastasizing leiomyoma: a case report and literature review. J Thorac Dis 2014 Jun;6(6):E92-8. doi: 10.3978/j.issn.2072-1439.2014.04.37.
26. Chen S, Zhang Y, Zhang J, Hu H, Cheng Y, Zhou J et al. Pulmonary benign metastasizing leiomyoma from uterine leiomyoma. World J Surg Oncol 2013 Jul 18;11:163. doi: 10.1186/1477-7819-11-163.
27. Chen YY, Wu ST, Hsu HH, Chen YC, Lee SC, Chang H et al. Pulmonary leiomyomas in a patient with bilateral renal cell cancer mimicking pulmonary metastases. Clin Imaging 2014 May-Jun;38(3):330-2. doi: 10.1016/j.clinimag.2014.01.008. Epub 2014 Feb 7.
28. Clark DH, Weed JC. Metastasizing leiomyoma: a case report. Am J Obstet Gynecol 1977;127(6):672-3.
29. Clément-Duchêne C, Vignaud JM,Regent D, Martinet Y. Benign metastsizing leiomyoma with lung cystis lesions and pneumothoraces: A case report. Respiratory Medicine CME3 2010;183-185.
30. [Cobellis L](http://www.ncbi.nlm.nih.gov/pubmed/?term=Cobellis%20L%5BAuthor%5D&cauthor=true&cauthor_uid=24772929), [Castaldi MA](http://www.ncbi.nlm.nih.gov/pubmed/?term=Castaldi%20MA%5BAuthor%5D&cauthor=true&cauthor_uid=24772929), [Mosca L](http://www.ncbi.nlm.nih.gov/pubmed/?term=Mosca%20L%5BAuthor%5D&cauthor=true&cauthor_uid=24772929), [Frega V](http://www.ncbi.nlm.nih.gov/pubmed/?term=Frega%20V%5BAuthor%5D&cauthor=true&cauthor_uid=24772929), [Ambrosio D](http://www.ncbi.nlm.nih.gov/pubmed/?term=Ambrosio%20D%5BAuthor%5D&cauthor=true&cauthor_uid=24772929), [Corvino F](http://www.ncbi.nlm.nih.gov/pubmed/?term=Corvino%20F%5BAuthor%5D&cauthor=true&cauthor_uid=24772929) et al. Benign pulmonary metastasizing leiomyomatosis: case report. [Eur J Gynaecol Oncol.](http://www.ncbi.nlm.nih.gov/pubmed/24772929) 2014;35(2):195-8.
31. Consamus EN, Reardon MJ, Ayala AG, Schwartz MR, Ro JY. Metastasizing leiomyoma to heart. Methodist Debakey Cardiovasc J 2014 Oct-Dec;10(4):251-4. doi: 10.14797/mdcj-10-4-251.
32. [de Ruiter GC](http://www.ncbi.nlm.nih.gov/pubmed/?term=de%20Ruiter%20GC%5BAuthor%5D&cauthor=true&cauthor_uid=17140158), [Scheithauer BW](http://www.ncbi.nlm.nih.gov/pubmed/?term=Scheithauer%20BW%5BAuthor%5D&cauthor=true&cauthor_uid=17140158), [Amrami KK](http://www.ncbi.nlm.nih.gov/pubmed/?term=Amrami%20KK%5BAuthor%5D&cauthor=true&cauthor_uid=17140158), [Spinner RJ](http://www.ncbi.nlm.nih.gov/pubmed/?term=Spinner%20RJ%5BAuthor%5D&cauthor=true&cauthor_uid=17140158). Benign metastasizing leiomyomatosis with massive brachial plexus involvement mimicking neurofibromatosis type 1. [Clin Neuropathol.](http://www.ncbi.nlm.nih.gov/pubmed/17140158) 2006 Nov-Dec;25(6):282-7.
33. Del Real-Romo ZJ, Montero-Cantú C, Villegas-Cabello O, Díaz-Elizondo JA, Reyes-Salas D, Palomo-Hoil R et al. Incidental benign metastasizing leiomyoma in a patient with bone sarcoma: a case report. Case Rep Surg 2014;2014:439061. doi: 10.1155/2014/439061. Epub 2014 Aug 26.
34. di Scioscio V, Feraco P, Miglio L, Toni F, Malvi D, Pacilli AM et al. Benign metastasizing leiomyoma of the lung: PET findings. J Thorac Imaging 2009 Feb;24(1):41-4.
35. [Drevelengas A](http://www.ncbi.nlm.nih.gov/pubmed/?term=Drevelengas%20A%5BAuthor%5D&cauthor=true&cauthor_uid=7713084), [Kalaitzoglou I](http://www.ncbi.nlm.nih.gov/pubmed/?term=Kalaitzoglou%20I%5BAuthor%5D&cauthor=true&cauthor_uid=7713084), [Sichletidis L](http://www.ncbi.nlm.nih.gov/pubmed/?term=Sichletidis%20L%5BAuthor%5D&cauthor=true&cauthor_uid=7713084). Benign pulmonary leiomyomatosis with cyst formation and breast metastasis: case report and literature review. [Eur J Radiol.](http://www.ncbi.nlm.nih.gov/pubmed/7713084) 1995 Jan;19(2):121-3.
36. Duncan SJ, Kadaria D. A Woman with a Lung Mass and Multiple Pulmonary Nodules. Am J Case Rep 2015 Jun 13;16:367-70. doi: 10.12659/AJCR.893749.
37. Egberts JH, Schafmayer C, Bauerschlag DO, Jänig U, Tepel J. Benign abdominal and pulmonary metastasizing leiomyoma of the uterus. Arch Gynecol Obstet 2006 Aug;274(5):319-22. Epub 2006 Apr 29.
38. Esteban JM, Allen WM, Schaerf RH. Benign metastasizing leiomyoma of the uterus: histologic and immunohistochemical characterization of primary and metastatic lesions. Arch Pathol Lab Med 1999;123(10):960-2.
39. Evans AJ, Wiltshaw E, Kochanowski SJ, Macfarlane A, Sears RT. Metastasizing leiomyoma of the uterus and hormonal manipulations. Case report. Brit J Obstet Gynaec 1986;93(6):646-8.
40. Fan D, Yi X. Pulmonary benign metastasizing leiomyoma: a case report. Int J Clin Exp Pathol 2014 Sep 15;7(10):7072-5.
41. Fatima S, Ahmed Z, Azam M. Benign metastasizing leiomyoma. Indian J Pathol Microbiol 2010 Oct-Dec;53(4):802-4.
42. Fu Y, Li H, Tian B, Hu B. Pulmonary benign metastasizing leiomyoma: a case report and review of the literature. World J Surg Oncol 2012 Dec 12;10:268. doi: 10.1186/1477-7819-10-268
43. Funakoshi Y, Sawabata N, Takeda S, Hayakawa M, Okumura Y, Maeda H. Pulmonary benign metastasizing leiomyoma from the uterus in a postmenopausal woman: report of a case. Surg Today 2004;34(1):55-7.
44. Gan MF, Lu HS. An undescribed coexistence of benign metastasizing leiomyoma in the lung with serous borderline tumor of the ovary. Eur J Gynaecol Oncol. 2013;34(2):193-5.
45. Galvin SD, Wademan B, Chu J, Bunton RW. Benign metastasizing leiomyoma: a rare metastatic lesion in the right ventricle. Ann Thorac Surg 2010 Jan;89(1):279-81. doi: 10.1016/j.athoracsur.2009.06.050.
46. Goto T, Maeshima A, Akanabe K, Hamaguchi R, Wakaki M, Oyamada Y et al. Benign metastasizing leiomyoma of the lung. Ann Thorac Cardiovasc Surg 2012;18(2):121-4. Epub 2011 Sep 29.
47. Goyle KK, Moore DF Jr, Garrett C, Goyle V. Benign metastasizing leiomyomatosis: case report and review. Am J Clin Oncol 2003 Oct;26(5):473-6.
48. Hafiz MA, Wang KP, Berkman A. Fine needle aspiration diagnosis of benign metastasizing leiomyoma of the lung. A case report. Acta Cytol 1994 May-Jun;38(3):398-402.
49. Hoetzenecker K, Ankersmit HJ, Aigner C, Lichtenauer M, Kreuzer S, Hacker S et al. Consequences of a wait-and-see strategy for benign metastasizing leiomyomatosis of the lung. Ann Thorac Surg 2009 Feb;87(2):613-4.
50. Huang H, Balos L, Chen F. Case report: Pulmonary Benign Metastasizing Leiomyoma Appears 26 Years after Resection of Uterine Leiomyoma. NAJ Med Sci 2012;5(1):55-57.
51. Jacobson TZ, Rainey EJ, Turton CWG. Pulmonary benign metastasising leiomyoma: response to treatment with goserelin. Thorax 1995;50(11):1225-1226.
52. Jautzke G, Müller-Ruchholtz E, Thalmann U. Immunohistological detection of estrogen and progesterone receptors in multiple and well differentiated leiomyomatous lung tumors in women with uterine leiomyomas (so-called benign metastasizing leiomyomas). A report on 5 cases. Pathol Res Pract 1996 Mar;192(3):215-23.
53. Jayakody S, Young K, Young B, Ferch R. Serial spread of benign metastasizing leiomyoma to the thoracic spine. J Clin Neurosci 2011 Aug;18(8):1135-7. doi: 10.1016/j.jocn.2011.01.004. Epub 2011 Jun 11.
54. Jeon HW, Choi SH, Sung SW, Park JK. Pulmonary benign metastasizing leiomyoma: report of three cases. World J Surg Oncol 2013 Oct 20;11:281. doi: 10.1186/1477-7819-11-281.
55. Jiang CY, Wang W, Yuan ZR. A rare pancreatic tumor in a 52-year-old Chinese woman. Pancreatic benign metastasizing leiomyoma. Gastroenterology 2013 Mar;144(3):511, 659-60. doi: 10.1053/j.gastro.2012.10.016. Epub 2013 Jan 17.
56. Guo-Qing J, Yu-Nong G, Min G, Hong Z, Xin Y, Wen W et al. Benign metastasizing leiomyoma: report of two cases and literature review. Chinese Medical Journal (English Edition) 2010;123(22):3367-3371.
57. Jo JH, Lee JH, Kim DC, Kim SH, Kwon HC, Kim JS et al. A case of benign metastasizing leiomyoma with multiple metastasis to the soft tissue, skeletal muscle, lung and breast. Korean J Intern Med 2006 Sep;21(3):199-201.
58. Jolissaint JS, Kilbourne SK, LaFortune K, Patel M, Lau CL. Benign metastasizing leiomyomatosis (BML): A rare cause of cavitary and cystic pulmonary nodules. Respir Med Case Rep 2015 Sep 21;16:122-4. doi: 10.1016/j.rmcr.2015.09.008.
59. Joo HJ, Han SS, Kwon JT, Park ES, Jung YY, Kim HK. Epidural intracranial metastasis from benign leiomyoma: a case report with literature review. Clin Neurol Neurosurg 2013 Jul;115(7):1180-3. doi: 10.1016/j.clineuro.2012.10.028.
60. Joseph V, Chacko G, Raghuram L, Rajshekhar V. Benign metastasizing leiomyoma causing spinal cord compression. Surg Neurol 2003 Dec;60(6):575-7; discussion 577-8.
61. [Kang MW](http://www.ncbi.nlm.nih.gov/pubmed/?term=Kang%20MW%5BAuthor%5D&cauthor=true&cauthor_uid=21353035), [Kang SK](http://www.ncbi.nlm.nih.gov/pubmed/?term=Kang%20SK%5BAuthor%5D&cauthor=true&cauthor_uid=21353035), [Yu JH](http://www.ncbi.nlm.nih.gov/pubmed/?term=Yu%20JH%5BAuthor%5D&cauthor=true&cauthor_uid=21353035), [Lim SP](http://www.ncbi.nlm.nih.gov/pubmed/?term=Lim%20SP%5BAuthor%5D&cauthor=true&cauthor_uid=21353035), [Suh KS](http://www.ncbi.nlm.nih.gov/pubmed/?term=Suh%20KS%5BAuthor%5D&cauthor=true&cauthor_uid=21353035), [Ahn JS](http://www.ncbi.nlm.nih.gov/pubmed/?term=Ahn%20JS%5BAuthor%5D&cauthor=true&cauthor_uid=21353035) et al. Benign metastasizing leiomyoma: metastasis to rib and vertebra. [Ann Thorac Surg.](http://www.ncbi.nlm.nih.gov/pubmed/21353035) 2011 Mar;91(3):924-6.
62. [Kayser K](http://www.ncbi.nlm.nih.gov/pubmed/?term=Kayser%20K%5BAuthor%5D&cauthor=true&cauthor_uid=11037349), [Zink S](http://www.ncbi.nlm.nih.gov/pubmed/?term=Zink%20S%5BAuthor%5D&cauthor=true&cauthor_uid=11037349), [Schneider T](http://www.ncbi.nlm.nih.gov/pubmed/?term=Schneider%20T%5BAuthor%5D&cauthor=true&cauthor_uid=11037349), [Dienemann H](http://www.ncbi.nlm.nih.gov/pubmed/?term=Dienemann%20H%5BAuthor%5D&cauthor=true&cauthor_uid=11037349), [André S](http://www.ncbi.nlm.nih.gov/pubmed/?term=Andr%C3%A9%20S%5BAuthor%5D&cauthor=true&cauthor_uid=11037349), [Kaltner H](http://www.ncbi.nlm.nih.gov/pubmed/?term=Kaltner%20H%5BAuthor%5D&cauthor=true&cauthor_uid=11037349) et al. Benign metastasizing leiomyoma of the uterus: documentation of clinical, immunohistochemical and lectin-histochemical data of ten cases. [Virchows Arch.](http://www.ncbi.nlm.nih.gov/pubmed/11037349) 2000 Sep;437(3):284-92.
63. Ki EY, Hwang SJ, Lee KH, Park JS, Hur SY. Benign metastasizing leiomyoma of the lung. World Journal of Surgical Oncology 2013;**11**:279. **DOI:** 10.1186/1477-7819-11-279.
64. Kim JJ, Park JK, Wang YP, Moon SW. Benign metastasising leiomyoma mistaken for pulmonary metastasis from an ovarian carcinoma. J Obstet Gynaecol 2015 Apr;35(3):325-6. doi: 10.3109/01443615.2014.954097. Epub 2014 Sep 10.
65. Koh DM, Burn PR, King DM. Benign metastasizing leiomyoma with intracaval leiomyomatosis. Br J Radiol 2000 Apr;73(868):435-7.
66. Kołaczyk K, Chamier-Ciemińska K, Walecka A, Chosia M, Szydłowska I, Starczewski A et al. Pulmonary benign metastasizing leiomyoma from the uterine leiomyoma: a case report. Pol J Radiol 2015 Feb 26;80:107-10. doi: 10.12659/PJR.892733.
67. Konis EE; Belsky RD. Metastasizing leiomyoma of the uterus. Report of a case. Obstet Gynecol 1966;27(3):442-6.
68. Kwon YI, Kim TH, Sohn JW, Yoon HJ, Shin DH, Park SS. Benign pulmonary metastasizing leiomvomatosis: case report and a review of the literature. Korean J Intern Med 2006 Sep;21(3):173-7.
69. Lazarous DG, Tsou E, Anderson E, O'Donnell AE. Benign metastasizing leiomyoma presenting with spontaneous pneumothorax. Chest 2005;128(4):488S-489S.
70. Lee HJ, Choi J, Kim KR. Pulmonary benign metastasizing leiomyoma associated with intravenous leiomyomatosis of the uterus: clinical behavior and genomic changes supporting a transportation theory. Int J Gynecol Pathol 2008 Jul;27(3):340-5. doi: 10.1097/PGP.0b013e3181656dab.
71. Lim SY, Park JC, Bae JG, Kim JI, Rhee JH. Pulmonary and retroperitoneal benign metastasizing leiomyoma. Clin Exp Reprod Med 2011 Sep;38(3):174-7. doi: 10.5653/cerm.2011.38.3.174. Epub 2011 Sep 30.

# Lin X, Fan W, Lang P, Hu Y, Zhang X, Sun X. Benign metastasizing leiomyoma identified using 18F-FDG PET/CT. Int J Gynaecol Obstet 2010 Aug;110(2):154-6. doi: 10.1016/j.ijgo.2010.03.017. Epub 2010 May 1.

1. Loukeri AA, Pantazopoulos IN, Tringidou R, Giampoudakis P, Valaskatzi A, Loukeri PA et al. Benign metastasizing leiomyoma presenting as cavitating lung nodules. Respir Care 2014 Jul;59(7):e94-7. doi: 10.4187/respcare.02775. Epub 2013 Nov 19.
2. Ma H, Cao J. Benign pulmonary metastasizing leiomyoma of the uterus: A case report. Oncol Lett 2015 Mar;9(3):1347-1350. Epub 2015 Jan 15.
3. Maltby JD, Misra D, Knight FH. 'Metastasizing leiomyoma' occurring as a mediastinal mass. Case report. Missouri Medicine 1980;77(6):304-6.
4. Maredia R, Snyder BJ, Harvey LA, Schwartz AM. Benign metastasizing leiomyoma in the lung. Radiographics 1998 May-Jun;18(3):779-82.
5. [Martin E](https://www.ncbi.nlm.nih.gov/pubmed/?term=Martin%20E%5BAuthor%5D&cauthor=true&cauthor_uid=6603116). Leiomyomatous lung lesions: a proposed classification. AJR Am J Roentgenol 1983 Aug;141(2):269-72.
6. Matsumoto T, Nonaka Y, Hayashi D, Kusumoto T. A case of a benign metastasizing leiomyoma which was diagnosed in 10 years after histerectomy. Asian J Endoscopic Surgery 2009;2(3):e192.

# [Miyamoto H](http://www.ncbi.nlm.nih.gov/pubmed/?term=Miyamoto%20H%5BAuthor%5D&cauthor=true&cauthor_uid=19291535), [Jones CE](http://www.ncbi.nlm.nih.gov/pubmed/?term=Jones%20CE%5BAuthor%5D&cauthor=true&cauthor_uid=19291535), [Raymond DP](http://www.ncbi.nlm.nih.gov/pubmed/?term=Raymond%20DP%5BAuthor%5D&cauthor=true&cauthor_uid=19291535), [Wandtke JC](http://www.ncbi.nlm.nih.gov/pubmed/?term=Wandtke%20JC%5BAuthor%5D&cauthor=true&cauthor_uid=19291535), [Strang JG](http://www.ncbi.nlm.nih.gov/pubmed/?term=Strang%20JG%5BAuthor%5D&cauthor=true&cauthor_uid=19291535), [Bourne PA](http://www.ncbi.nlm.nih.gov/pubmed/?term=Bourne%20PA%5BAuthor%5D&cauthor=true&cauthor_uid=19291535) et al. Pulmonary metastases from uterine neoplasms after long tumour-free interval: four cases and review of the literature. [Pathology.](http://www.ncbi.nlm.nih.gov/pubmed/19291535) 2009;41(3):234-41.

1. Miyazaki M, Nakayama A, Noda D, Maehara Y, Tsushima Y. Difficulty in complete transarterial embolization for pulmonary benign metastasizing leiomyoma with massive hemoptysis. Jpn J Radiol 2014 Jan;32(1):53-7. doi: 10.1007/s11604-013-0266-9.
2. Mizuno M, Nawa A, Nakanishi T, Yatabe Y. Clinical benefit of endocrine therapy for benign metastasizing leiomyoma. Int J Clin Oncol 2011 Oct;16(5):587-91. doi: 10.1007/s10147-010-0156-4.
3. [Mlika M](http://www.ncbi.nlm.nih.gov/pubmed/?term=Mlika%20M%5BAuthor%5D&cauthor=true&cauthor_uid=19771765), [Ayadi-Kaddour A](http://www.ncbi.nlm.nih.gov/pubmed/?term=Ayadi-Kaddour%20A%5BAuthor%5D&cauthor=true&cauthor_uid=19771765), [Smati B](http://www.ncbi.nlm.nih.gov/pubmed/?term=Smati%20B%5BAuthor%5D&cauthor=true&cauthor_uid=19771765), [Ismaïl O](http://www.ncbi.nlm.nih.gov/pubmed/?term=Isma%C3%AFl%20O%5BAuthor%5D&cauthor=true&cauthor_uid=19771765), [El Mezni F](http://www.ncbi.nlm.nih.gov/pubmed/?term=El%20Mezni%20F%5BAuthor%5D&cauthor=true&cauthor_uid=19771765). Benign metastasizing leiomyoma: report of 2 cases and review of the literature. [Pathologica.](http://www.ncbi.nlm.nih.gov/pubmed/19771765) 2009 Feb;101(1):9-11.
4. Mogi A, Hirato J, Kosaka T, Yamaki E, Kuwano H. Benign metastasizing leiomyoma of the lung: report of a case. Gen Thorac Cardiovasc Surg 2013 Dec;61(12):719-22. doi: 10.1007/s11748-012-0174-4.
5. [Moon H](http://www.ncbi.nlm.nih.gov/pubmed/?term=Moon%20H%5BAuthor%5D&cauthor=true&cauthor_uid=19506459), [Park SJ](http://www.ncbi.nlm.nih.gov/pubmed/?term=Park%20SJ%5BAuthor%5D&cauthor=true&cauthor_uid=19506459), [Lee HB](http://www.ncbi.nlm.nih.gov/pubmed/?term=Lee%20HB%5BAuthor%5D&cauthor=true&cauthor_uid=19506459), [Kim SR](http://www.ncbi.nlm.nih.gov/pubmed/?term=Kim%20SR%5BAuthor%5D&cauthor=true&cauthor_uid=19506459), [Choe YH](http://www.ncbi.nlm.nih.gov/pubmed/?term=Choe%20YH%5BAuthor%5D&cauthor=true&cauthor_uid=19506459), [Chung MJ](http://www.ncbi.nlm.nih.gov/pubmed/?term=Chung%20MJ%5BAuthor%5D&cauthor=true&cauthor_uid=19506459) et al. Pulmonary benign metastasizing leiomyoma in a postmenopausal woman. [Am J Med Sci.](http://www.ncbi.nlm.nih.gov/pubmed/19506459) 2009 Jul;338(1):72-4.
6. Naito M, Kobayashi T, Yoshida M, Fujiwara K, Onishi M, Fujiwara A et al. Solitary pulmonary nodule of benign metastasizing leiomyoma associated with primary lung cancer: a case report. J Med Case Rep 2011 Oct 5;5:500. doi: 10.1186/1752-1947-5-500.
7. Nakajo M, Nakayama H, Sato M, Fukukura Y, Nakajo M, Kajiya Y et al. FDG-PET/CT finding of benign metastasizing leiomyoma of the lung. Acta Radiol Short Rep 2012 Apr 23;1(3). pii: arsr.2012.120012. doi: 10.1258/arsr.2012.120012.
8. Nasu K, Tsuno A, Takai N, Narahara H. A case of benign metastasizing leiomyoma treated by surgical castration followed by an aromatase inhibitor, anastrozole. Arch Gynecol Obstet 2009 Feb;279(2):255-7. doi: 10.1007/s00404-008-0698-0.
9. [Nucci MR](http://www.ncbi.nlm.nih.gov/pubmed/?term=Nucci%20MR%5BAuthor%5D&cauthor=true&cauthor_uid=17460458), [Drapkin R](http://www.ncbi.nlm.nih.gov/pubmed/?term=Drapkin%20R%5BAuthor%5D&cauthor=true&cauthor_uid=17460458), [Dal Cin P](http://www.ncbi.nlm.nih.gov/pubmed/?term=Dal%20Cin%20P%5BAuthor%5D&cauthor=true&cauthor_uid=17460458), [Fletcher CD](http://www.ncbi.nlm.nih.gov/pubmed/?term=Fletcher%20CD%5BAuthor%5D&cauthor=true&cauthor_uid=17460458), [Fletcher JA](http://www.ncbi.nlm.nih.gov/pubmed/?term=Fletcher%20JA%5BAuthor%5D&cauthor=true&cauthor_uid=17460458). Distinctive cytogenetic profile in benign metastasizing leiomyoma: pathogenetic implications. [Am J Surg Pathol.](http://www.ncbi.nlm.nih.gov/pubmed/17460458) 2007 May;31(5):737-43.
10. Ogawa M, Hara M, Ozawa Y, Moriyama S, Yano M, Shimizu S et al. Benign metastasizing leiomyoma of the lung with malignant transformation mimicking mediastinal tumor. Clin Imaging 2011 Sep-Oct;35(5):401-4. doi: 10.1016/j.clinimag.2010.11.003.

# [Okabe R](http://www.ncbi.nlm.nih.gov/pubmed/?term=Okabe%20R%5BAuthor%5D&cauthor=true&cauthor_uid=25360407), [Shoji T](http://www.ncbi.nlm.nih.gov/pubmed/?term=Shoji%20T%5BAuthor%5D&cauthor=true&cauthor_uid=25360407), [Huang CL](http://www.ncbi.nlm.nih.gov/pubmed/?term=Huang%20CL%5BAuthor%5D&cauthor=true&cauthor_uid=25360407). Benign metastasizing leiomyoma of the lung with spontaneous pneumothorax. [Thorac Cardiovasc Surg Rep.](http://www.ncbi.nlm.nih.gov/pubmed/25360407) 2013 Dec;2(1):26-8.

1. Orejola WC, Vaidya AP, Elmann EM. Benign metastasizing leiomyomatosis of the lungs presenting a miliary pattern. Ann Thorac Surg 2014 Nov;98(5):e113-4. doi: 10.1016/j.athoracsur.2014.07.057.
2. Parenti DJ, Morley TF, Giudice JC. Benign metastasizing leiomyoma. A case report and review of the literature. [Review] [10 refs][Erratum appears in Respiration 1993;60(2):136]Respiration. 1992;59(6):347-50.
3. Pekçolaklar A, Metin M, Çıtak N, Gürses A. Pulmonary benign metastasizing leiomyoma from the uterus in a postmenopausal woman: report of case. Indian J Thorac Cardiovasc Surg 2011; 27:50–52. DOI 10.1007/s12055-010-0067-z.
4. Pérez-Ferrer P, Chiner E, Sancho-Chust JN, Arlandis M. Pulmonary benign metastasizing leiomyoma, a rare cause of pulmonary nodules. Arch Bronconeumol 2016 Apr;52(4):226-7. doi: 10.1016/j.arbres.2015.08.004.
5. Ponea AM, Marak CP, Goraya H, Guddati AK. Benign metastatic leiomyoma presenting as a hemothorax. Case Rep Oncol Med 2013;2013:504589. doi: 10.1155/2013/504589.
6. [Poujade O](http://www.ncbi.nlm.nih.gov/pubmed/?term=Poujade%20O%5BAuthor%5D&cauthor=true&cauthor_uid=21077481), [Genin AS](http://www.ncbi.nlm.nih.gov/pubmed/?term=Genin%20AS%5BAuthor%5D&cauthor=true&cauthor_uid=21077481), [Dhouha M](http://www.ncbi.nlm.nih.gov/pubmed/?term=Dhouha%20M%5BAuthor%5D&cauthor=true&cauthor_uid=21077481), [Luton D](http://www.ncbi.nlm.nih.gov/pubmed/?term=Luton%20D%5BAuthor%5D&cauthor=true&cauthor_uid=21077481). A benign metastasizing leiomyoma involving a nodule in the pulmonary artery: case and literature review. [Eur J Gynaecol Oncol.](http://www.ncbi.nlm.nih.gov/pubmed/21077481) 2010;31(3):329-32.
7. Radzikowska E, Szczepulska-Wójcik E, Langfort R, Oniszh K, Wiatr E. Benign pulmonary metastasizing leiomyoma uteri. Case report and review of literature. Pneumonol Alergol Pol 2012;80(6):560-4.
8. Rakhshani N, Hormazdi M, Abolhasani M, Shahzadi M. Benign metastasizing leiomyoma of the uterus. Arch Iran Med 2007 Jan;10(1):97-9.
9. Rao AV, Wilson J, Sylvester K. Pulmonary benign metastasizing leiomyoma following hysterectomy: a clinicopathologic correlation. J Thorac Oncol 2008 Jun;3(6):674-6. doi: 10.1097/JTO.0b013e3181757a46.
10. Regueiro P, Paredes LA, Castellarnau M, Sastre ME, Fernandez S, Cayuela E. Benign metastasising leiomyoma: a case report of lung and pleural metastases. Ultrasound Obst Gyn 2014;44(1):181-369.
11. Sapmaz F, Ergin M, Katrancioglu O, Gonlugur T, Gonlugur U, Elagoz S. Benign Metastasizing Leiomyoma. Lung 2008;186(4):271-273.
12. Sarici F, Babacan T, Altundag K, Balakan O, Gullu I. Successful treatment of benign metastasizing leiomyoma with oral alternated chemotherapeutic agents. J BUON 2013 Jul-Sep;18(3):799.
13. Seghal R, Maghrabi A. Benign metastasizing leiomyoma: ‘a sheep in wolf’s clothing’. Commun Oncol 2013;10:122-125.
14. Scutiero G, Nappi L, Spada A, Bufo P, Greco P. Benign metastasizing leiomyoma of external iliac vessels: an unusual case report. Eur J Obstet Gyn R B 2011;154(1):114-5.
15. [Shariftabrizi A](http://www.ncbi.nlm.nih.gov/pubmed/?term=Shariftabrizi%20A%5BAuthor%5D&cauthor=true&cauthor_uid=26244194), [Abdullah A](http://www.ncbi.nlm.nih.gov/pubmed/?term=Abdullah%20A%5BAuthor%5D&cauthor=true&cauthor_uid=26244194), [Jacob S](http://www.ncbi.nlm.nih.gov/pubmed/?term=Jacob%20S%5BAuthor%5D&cauthor=true&cauthor_uid=26244194), [Molin A](http://www.ncbi.nlm.nih.gov/pubmed/?term=Molin%20A%5BAuthor%5D&cauthor=true&cauthor_uid=26244194), [Panarelli E](http://www.ncbi.nlm.nih.gov/pubmed/?term=Panarelli%20E%5BAuthor%5D&cauthor=true&cauthor_uid=26244194), [Samuelson R](http://www.ncbi.nlm.nih.gov/pubmed/?term=Samuelson%20R%5BAuthor%5D&cauthor=true&cauthor_uid=26244194) et al. Incidental Finding of Synchronous, Benign, Metastasizing Leiomyoma with Distinct Cytogenetics in the Lung and Uterus. [Conn Med.](http://www.ncbi.nlm.nih.gov/pubmed/26244194) 2015 Jan;79(1):37-9.
16. Shin MS, Fulmer JD, Ho KJ. Unusual computed tomographic manifestations of benign metastasizing leiomyomas as cavitary nodular lesions or interstitial lung disease. Clin Imaging 1996 Jan-Mar;20(1):45-9.
17. Silva I, Tomé V, Oliveira J. Benign metastasising leiomyoma: a progressive disease despite chemical and surgical castration. BMJ Case Rep 2012 Mar 27;2012. pii: bcr0120125505. doi: 10.1136/bcr.01.2012.5505
18. [Simon P](http://www.ncbi.nlm.nih.gov/pubmed/?term=Simon%20P%5BAuthor%5D&cauthor=true&cauthor_uid=21337844), [Dept S](http://www.ncbi.nlm.nih.gov/pubmed/?term=Dept%20S%5BAuthor%5D&cauthor=true&cauthor_uid=21337844), [Lefranc F](http://www.ncbi.nlm.nih.gov/pubmed/?term=Lefranc%20F%5BAuthor%5D&cauthor=true&cauthor_uid=21337844), [Noel JC](http://www.ncbi.nlm.nih.gov/pubmed/?term=Noel%20JC%5BAuthor%5D&cauthor=true&cauthor_uid=21337844). Brain metastasis after breast cancer and hysterectomy for a benign leiomyoma. [Acta Chir Belg.](http://www.ncbi.nlm.nih.gov/pubmed/21337844) 2010 Nov-Dec;110(6):611-3.
19. Stephenson CA, Henley FT, Goldstein AR. Benign metastasizing leiomyoma. Ala J Med Sci 1984 Jan;21(1):78-81.
20. Taftaf R, Starnes S, Wang J, Shipley R, Namad T, Khaled R et al. Benign metastasizing leiomyoma: a rare type of lung metastases-two case reports and review of the literature. Case Rep Oncol Med 2014;2014:842801. doi: 10.1155/2014/842801.
21. Takemura G, Takatsu Y, Kaitani K, Ono M, Ando F, Tanada S et al. Metastasizing uterine leiomyoma. A case with cardiac and pulmonary metastasis. Pathol Res Pract 1996 Jun;192(6):622-9; discussion 630-3.
22. [Tatebe S](http://www.ncbi.nlm.nih.gov/pubmed/?term=Tatebe%20S%5BAuthor%5D&cauthor=true&cauthor_uid=19330762), [Oka K](http://www.ncbi.nlm.nih.gov/pubmed/?term=Oka%20K%5BAuthor%5D&cauthor=true&cauthor_uid=19330762), [Kuraoka S](http://www.ncbi.nlm.nih.gov/pubmed/?term=Kuraoka%20S%5BAuthor%5D&cauthor=true&cauthor_uid=19330762), [Yatabe Y](http://www.ncbi.nlm.nih.gov/pubmed/?term=Yatabe%20Y%5BAuthor%5D&cauthor=true&cauthor_uid=19330762). Benign metastasizing leiomyoma of the lung: potential role of low-grade malignancy. [Thorac Cardiovasc Surg.](http://www.ncbi.nlm.nih.gov/pubmed/19330762) 2009 Apr;57(3):180-3.
23. Teixeira BC, Mahfouz K, Escuissato DL, Costa AF, Noronha Ld. Solitary benign metastasizing leiomyoma: imaging features and pathological findings. J Bras Pneumol 2014 Mar-Apr;40(2):193-5.
24. Tohya T, Tajima T, Takeshita Y, Ito K, Kuriwaki K, Katabuchi H. Case of concurrent benign metastasizing leiomyoma in the lung and retroperitoneum, with a focus on its etiology. J Obstet Gynaecol Res 2014 Aug;40(8):2010-3. doi: 10.1111/jog.12424.
25. Tori M, Akamatsu H, Mizutani S, Yoshidome K, Oyama T, Ueshima S et al. Multiple benign metastasizing leiomyomas in the pelvic lymph nodes and biceps muscle: report of a case. Surg Today 2008;38(5):432-5. doi: 10.1007/s00595-007-3609-2.
26. Usman Y, Ishaq M, Awab A, Palacios M. Pulmonary benign metastasizing leiomyoma, why metastsizes if benign. Chest 2015;148(4):627A. Doi: 10.1378/chest.2264285.
27. Wang LX, Lv FZ, Ma X, Jiang JY. Multifocal Osteolytic Lesions Within Lumbar Spine in a Middle-Aged Chinese Woman: A Benign Metastasizing Leiomyoma? Spine 2012;37(4):E259-E263.
28. [Wei WT](https://www.ncbi.nlm.nih.gov/pubmed/?term=Wei%20WT%5BAuthor%5D&cauthor=true&cauthor_uid=26171020), [Chen PC](https://www.ncbi.nlm.nih.gov/pubmed/?term=Chen%20PC%5BAuthor%5D&cauthor=true&cauthor_uid=26171020). Benign metastasizing leiomyoma of the lung: A case report and literature review. [Oncol Lett.](https://www.ncbi.nlm.nih.gov/pubmed/?term=wei%2C+chen+benign+metastasizing) 2015 Jul;10(1):307-312. Epub 2015 May 19.
29. Wentling GK, Sevin BU, Geiger XJ, Bridges MD. Benign metastasizing leiomyoma responsive to megestrol: case report and review of the literature. Int J Gynecol Cancer 2005 Nov-Dec;15(6):1213-7.
30. Wiencek-Weiss AJ, Bruliński K. **Benign metastasizing leiomyomas in the lungs: a case study.** Kardiochir Torakochi 2016; 13 (1): 61-63. DOI: **10.5114/kitp.2016.58970.**
31. Winkler TR, Burr LH, Robinson CL. Benign metastasizing leiomyoma. Ann Thorac Surg 1987 Jan;43(1):100-1.
32. Wongsripuemtet J, Ruangchira-Urai R, Stern EJ, Kanne JP, Muangman N. Benign metastasizing leiomyoma. J Thorac Imaging 2012 Mar;27(2):W41-3. doi: 10.1097/RTI.0b013e318215cc26.
33. Xiao H, Li B, Li W, Feng X, Wu L. A rare case of benign abdominal wall and pelvic metastasizing leiomyomas following hysterectomy. J Obstet Gynaecol 2012 Feb;32(2):198-9.
34. Yoon G, Kim TJ, Sung CO, Choi CH, Lee JW, Lee JH et al. Benign metastasizing leiomyoma with multiple lymph node metastasis: a case report. Cancer Res Treat 2011 Jun;43(2):131-3. doi: 10.4143/crt.2011.43.2.131.
35. Yu R, [Ferri M](https://www.ncbi.nlm.nih.gov/pubmed/?term=Ferri%20M%5BAuthor%5D&cauthor=true&cauthor_uid=25802769). An unusual cause of pulmonary nodules in the emergency department. Case Rep Emerg Med. 2015;2015:278020. doi: 10.1155/2015/278020.
36. Zhang Y, Clark LH, Sheng X, Zhou C. Successful en bloc venous resection with reconstruction and subsequent radiotherapy for 2 consecutive recurrences of intravenous leiomyoma- a case report. BMC Cancer 2016 Jan 6;16:6. doi: 10.1186/s12885-015-2045-8.
